# Manual, *In situ*, Real-Time Nanofabrication using Cracking through Indentation

**DOI:** 10.1038/srep18892

**Published:** 2016-01-04

**Authors:** Koo Hyun Nam, Young D. Suh, Junyeob Yeo, Deokha Woo

**Affiliations:** 1 Department of Physics, Kyunghee University, Seoul 02447, Korea; 2 Sensor System Research Center, Korea Institute of Science and Techonology, Seoul 02792, Korea; 3 Department of Mechanical Engineering, Seoul National University, Seoul 08826, Korea

## Abstract

Nanofabrication has seen an increasing demand for applications in many fields of science and technology, but its production still requires relatively difficult, time-consuming, and expensive processes. Here we report a simple but very effective one dimensional (1D) nano-patterning technology that suggests a new nanofabrication method. This new technique involves the control of naturally propagating cracks initiated through simple, manually generated indentation, obviating the necessity of complicated equipment and elaborate experimental environments such as those that employ clean rooms, high vacuums, and the fastidious maintenance of processing temperatures. The channel fabricated with this technique can be as narrow as 10 nm with unlimited length and very high cross-sectional aspect ratio, an accomplishment difficult even for a state-of-the-art technology such as e-beam lithography. More interestingly, the fabrication speed can be controlled and achieved to as little as several hundred micrometers per second. Along with the simplicity and real-time fabrication capability of the technique, this tunable fabrication speed makes the method introduced here the authentic nanofabrication for *in situ* experiments.

Given the maturation of current nanofabrication technologies, including e-beam^1^, focused ion beam (FIB)^2^, and nanoimprint lithographies^3^, the creation of structures on scales of tens of nanometers is no longer surprising. 1D nanostructures such as nanochannels especially have been used with increasing frequency in various fields including electronics, chemistry, and the life sciences. With such developments, the domain of related researches has expanded down to the nanoscale, and the need for nanostructure fabrication has increased commensurately. However, the generation of nanostructures for such purposes is currently restricted by the limitations of the few technologies presently available^4^. And despite the continued development of these technologies, breakthroughs which would overcome their problems and limitations have not yet emerged^5^. Additionally, conventional nanofabrication technologies are limited by processing time and fabrication cost as well as by the difficulties of maintaining clean, high vacuum or fastidious temperature environments.

Especially on the nanometer scale, cracks have been studied as an interesting tiny structure^6^,^7^,^8^ as a candidate for nanoscale pattern former^9^. However, not much attention and effort have drawn to the utilization of such structures because precise and reliable control is extremely difficult^10^,^11^, and previous attempts^6^,^12^,^13^ reveals that the realization of controlled cracking has heretofore been a complicated and laborious task. Nevertheless, cracking as an atomic-scale phenomenon^14^ is anticipated to be a fabrication technology as one of few applicable options for the scale down to sub-nanometer scales.

In this study, we describe a completely new approach to the fabrication of nanostructures created by intentional and controlled (although naturally propagating) occurrences of cracking. This technique makes it possible to produce nanostructures readily by hand with an easily constructed, mass-producible platform and basic inexpensive tools. The process is simple enough to allow even those without engineering backgrounds or special facilities to fabricate controlled crack structures similar to nanochannels in specific orientation down to 10 nanometers. All that is required is the ready-made platform and an inexpensive indentation tool such as a tungsten carbide pencil.

## Results

### Platform preparation and basic manual-control of crack

**Figure 1:**
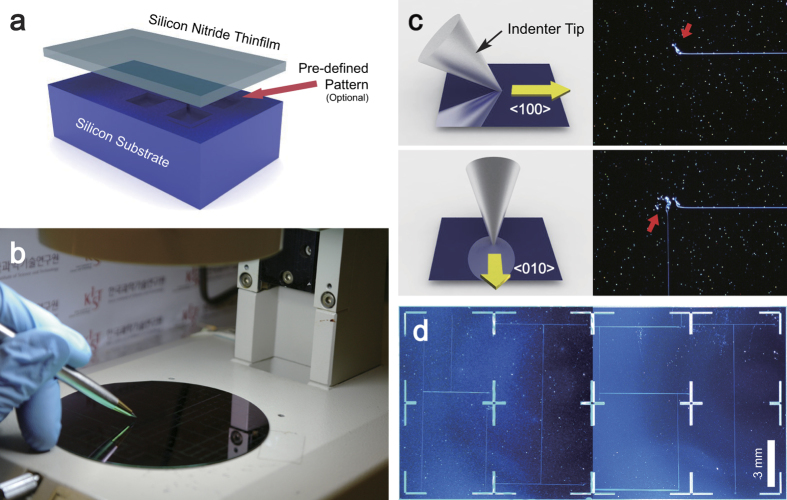
Crack initiated by indentation and controlled in a Si_3_N_4_ film and Si wafer system. (**a**) Sample preparation consisting of the LPCVD deposition of a Si_3_N_4_ film of 700~1100 nm in thickness on a Si wafer with optional predefined patterns etched into the substrate prior to film deposition. (**b**) A channel-like nanostructures easily built using cracking induced by indentation. Control is achieved through the manual manipulation of a sufficiently hard indenting implement. (**c**) Direction control of cracking in the indentation process. (**d**) Nanostructure fabrication using indentation on a prepatterned platform to write the letters, “*KIST”*. Bright structures in the figure are crack-stop structures which have been prepatterned to arrest propagating cracks, and this particular platform design is intended to generate complicated nanopatterns with a freedom similar to the seven-segment displays used in the field of electronics. The thickness of the Si_3_N_4_ film in the left half of the image is 700 nm, and that of right half is 800 nm. The nanopatterns for each half are approximately 20 and 25 nm in width, respectively, and tens of millimeters in length.

In order to achieve a readily available manual nanofabrication method, development and material process of a platform that allows elaborate crack control by indentation is a crucial step. Despite its usefulness and functionality, the preparation of samples for this study is simple and straightforward. As shown in Fig. 1a, the sole process required is the construction of the platform through the deposition of a silicon nitride (Si_3_N_4_) film of 700~1100 nm onto a single crystal silicon (Si) wafer of 525 *μ*m in thickness. The Si_3_N_4_ film was deposited by low pressure chemical vapor deposition (LPCVD) process, which imparts very high tensile stress (~1.3 GPa) to the Si_3_N_4_/Si system^15^. Along with the fact that the Si_3_N_4_ film has a fracture energy appropriately higher than that of Si substrate, the high tensile stress induced by the LPCVD process makes the Si_3_N_4_/Si combination one of the few possible bi-material candidates for such applications^16^. In order to permit additional modifications that take advantage of the Si wafer surface’s cracking propensities, optional processing prior to deposition of the Si_3_N_4_ film is possible through the prepatterning of microfabrications, including the addition of crack-stop structures for tailoring crack length (see also Supplementary Fig. S1). Following the preparation of the platform, the sample constitutes a medium upon which a crack is readily generated through indentation by any sufficiently hard implement. From the user’s standpoint, this technique is fast, very easy, and can be realized with inexpensive, readily available, and easy-to-use tools such as tungsten carbide pencils on the separately constructed platform. Additional advantage for this approach is that platform can be made in advance in multiple quantities or provided by other facilities where a technical comparative advantage is held. Although a more precise positioning and controlling of cracks is possible with engineered tools such as micro/nanoindenters^17^, to demonstrate the feasibility and ease of these readily accessible nanoscale manual fabrication techniques, even under rudimentary experimental conditions, all the work presented here was deliberately conducted by hand with a tungsten carbide pencil (general-purpose glass scribing tool).

Cracks in Si_3_N_4_ film deposited on a (100) Si wafer have strong tendency to propagate in the <100> direction due to the crystallographic anisotropy of the substrate^12^,^18^. As demonstrated in Fig. 1b, this tendency may be exploited to create a nanoscale controlled cracks through indentation with a handheld tungsten carbide pencil, obviating the need for complicated and expensive process. This strong tendency prevents a crack from initiating through any orientation other than that in the <100> direction; moreover, even if the crack direction is at an uncommon orientation, which occasionally occurs, it is soon forced to kink to correct its route to a favorable orientation, thus increasing the controllability of the crack orientation at some points. As demonstrated in Fig. 1c, crack initiation and orientation are easily and precisely manipulated though the careful manual but otherwise unassisted, control of the implement. Gently placing the indenter tip at the intended position of one end of the crack and pushing the tip in the desired direction is sufficient to fabricate a channel-like nanostructure. The minimum force required to initiate a crack only applies to newly opened cut surfaces, and the amount of force should be appropriate, as the application of excessive force may result in the generation of additional unwanted cracks. The desired orientation has been chosen from one of the <100> direction, which represent four orientations each at 90 degrees respective to the others, as illustrated in Fig. 1c. Crack generation and full control of crack direction with 100% yield can be attained with a little practice, even by a person with no previous experience of this or any other microfabrication technique (see also Supplementary Fig. S2 for an example experiment with an unskilled high school student conducting nanoscale pattern fabrication using this technique). More than two cracks having different <100> orientations can be generated concurrently when force is applied onto the indenter tip toward in other than the <100> direction; however, the repeatability is not as high as it is in the generation of a single crack generated aligned in the <100> orientation. Once initiated by manual indentation, the crack continues to propagate naturally across the platform’s uniform Si_3_N_4_ film; and this makes possible the production of very long nanostructures limited only by the dimensions of the substrate wafer. The uniformly distributed residual stress of the film constitutes a self-sustaining fracture dynamics, which in ideal circumstances would allow the crack to propagate perpetually. For the same reason, the shape of the indenter tip and the initial crack size do not affect the geometry of a propagating crack which is only dependent on the residual stress in the sample of the Si_3_N_4_/Si system.

The crack is arrested either by an encounter with a discontinuity in the film or, as in Supplementary Fig. S1, through the interposition of a crack-stop structure. Figure 1d shows the inscription of the letters “*KIST*” in very long 20 and 25 nm wide nanostructures that easily extend for tens of millimeters, a result that would be difficult using other current nanofabrication technologies. As shown in Fig. 1d, the unique feature of this technique is that the nanostructure created by the approach is clearly visible through optical microscopy or even to the unaided eye under illumination (optionally with appropriate tilting of the sample or light source; see also Supplementary Fig. S3). Note that within the deep channel-like geometry light scattering makes the crack visible, despite the theoretical impossibility of an optical observation of features of such small size (<<diffraction-limit of light). Although non-uniform scattering may occur according to large-area observations under a microscope, as shown in Fig. 1d, sets of observations confirmed the uniformity of the width of the crack, as shown in Fig. S4. The actual visibility of the pattern generated by the artifact’s light scattering, means that the technique can be undertaken without magnification, and the controlled production of artifacts at this scale is a feat never before reported for a true hand-tooled small patterning technology.

### Mechanics of controlled cracking and immediate applications

**Figure 2:**
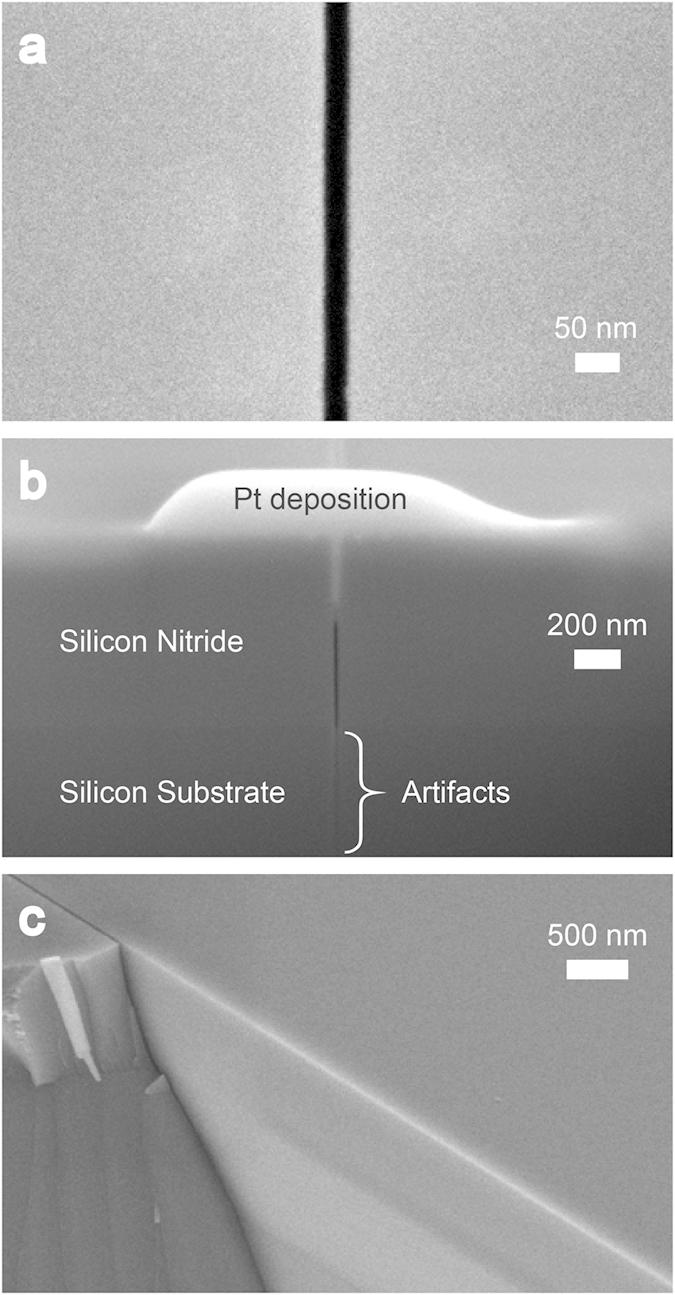
Geometry of nanostructure fabricated by cracking. Scanning electron microscopy (SEM) image showing that crack propagation in the Si_3_N_4_ film is (**a**) very straight, and (**b**) vertical toward the Si substrate interface. (**b**) is a cross-sectional view milled by a FIB. The blurred vertical line in (**b**), which runs into the Si substrate, is an FIB artifact known as “curtaining”^37^. (**c**) SEM image of the nanostructure’s clean side wall which is a newly-created cutting plane of cracking. One half of the cracked film is exposed by manual cleaving.

Our research results indicate that residual stress between the substrate and film, which determines the mechanics of cracking, rises as the thickness of the Si_3_N_4_ layer increases, and consequently, the speed of propagation also increases. The thickness values of the films in Si_3_N_4_ film/Si substrate system used in this study were tested between 500 and 1100 nm at 100 nm intervals, and it was possible to produce cracks by indentation for thickness values greater than 600 nm. Theoretically, the critical thickness for the continuation of crack propagation through film is defined by 
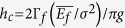
, where 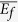 is the material plane strain tensile modulus which is given by 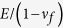. Γ_*f*_, *ν*_*f*_, and *g* are, respectively, the fracture energy and Poison’s ratio of the film, and nondimensionalized crack opening displacement determined by material properties^19^,^20^,^21^. Since the residual stress in the formula is the only parameter affected by the variation of the Si_3_N_4_ film’s thickness, the consequence of thickening Si_3_N_4_ film is the decrease of *h*_*c*_; thus the crack propagation is assured as long as the Si_3_N_4_ film thickness is greater than 600 nm in the system. The channeling crack can accompany substrate penetration under certain conditions. Our experimental results including the one shown in Supplementary Fig. S5 obey the theoretical prediction^19^,^21^ (see Supplementary Information for further discussion of substrate penetration of channeling cracking). As shown in Fig. 2a,b, the cracking forms a channeling crack in layered materials^20^ of Si_3_N_4_/Si system, and has produced a very straight channel characterized by a clean vertical break running normal to the film’s surface plane. Along with the high aspect ratio in the cross-sectional view, this atomic-scale clean geometry (Fig. 2c) is typical for brittle cracking, and an attractive feature uniquely achievable through this technique.

**Figure 3:**
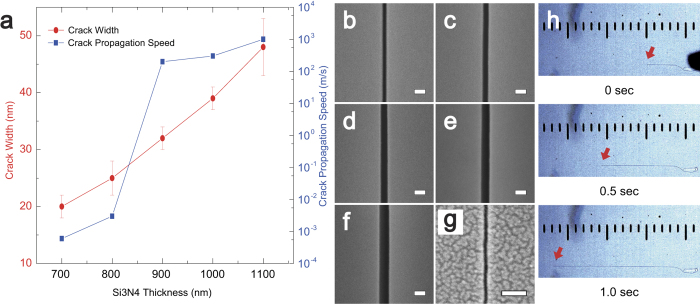
Mechanics of cracking. (**a**) The variations of crack width and propagation speed in terms of the thickness of the Si_3_N_4_ film. (**b**–**g**) SEM images of samples showing the variations of crack width in terms of the thickness of the Si_3_N_4_ film. The thickness values of the films are 700, 800, 900, 1000, 1100, and 650 nm, respectively. The scale bar indicates 50 nm. The width of sample (**g**) is 8 nm, achieved by chemical thinning of the Si_3_N_4_ film down to approximately 650 nm in thickness. (**h**) The propagating crack captured at 0.5 second intervals in a Si_3_N_4_ film of 700 nm in thickness. Each scale division indicates 50 *μ*m.

The width of cracks and the speed of propagation can be precisely defined by the thickness of the Si_3_N_4_ film. As shown in Fig. 3a, the increase of crack width is nearly proportional to Si_3_N_4_ thickness between 700 nm and 1100 nm. The sample-to-sample thickness uniformity of Si_3_N_4_ film within a sample used in this study is ±20 nm. On the contrary, the crack width variation among samples (within-sample uniformity) that have similar Si_3_N_4_ thickness is ±2~3 nm except for the samples with the thickness greater than 1000 nm. It is anticipated that the instability induced at the greater thickness is the cause of the greater crack width variation. Therefore, nanostructures with any particular width can be fabricated through accurate control of the Si_3_N_4_ film deposition as shown in Fig. 3b–g. Figure 3g, illustrating a nanochannel of approximately 8 nm in width, is not involved in the statistics of Fig. 3a because it is achieved by chemical thinning of the Si_3_N_4_ film down to approximately 650 nm in thickness. These experiment results shows that the channel width, *δ*, increases as 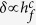, where *h*_*f*_ is the thickness of the Si_3_N_4_ film, and *c* is a number slightly greater than 1. It is in agreement with the theoretical crack opening displacement derived from 
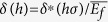
, where δ^*^ is a nondimensionalized crack opening displacement at the top edge, determined by material properties^20^. This indicates that the contribution of the increase of the residual stress to the width of the crack is not as significant as that of the thickness variation of the film.

As shown in Fig. 3a, the crack propagation speed increases as Si_3_N_4_ thickness increases. An especially sharp crack propagation speed increase has been found in the thickness regime between 800 nm and 900 nm Si_3_N_4_. The fastest crack propagation speed measured with ordinary equipment at thickness of 1100 nm exceeds 1000 m/s. As shown in Fig. 3h, the speed of crack propagation is slowest (600 *μ*m/sec) for the thinnest configuration (a Si_3_N_4_ film of 700 nm in thickness; see also Supplementary Video S1). Considering speed dependent or sensitive parameters in various experiments, the especially low speed of crack propagation in a thin Si_3_N_4_ film as compared to uncontrollable and unmeasurable fabrication speed of the products with conventional equipments may suggest that the nanochannel fabrication demonstrated in this study is a suitable nanofabrication method to be employed in *in situ* experiments^22^. As the thickness of the Si_3_N_4_ increases greater than approximately 1000 nm, the stability of generating crack starts to drop significantly in accordance with the increase of the crack propagation speed^10^,^23^. Consequently, the control of cracking in a sample with the Si_3_N_4_ thicker than 1100 nm is difficult to be achieved through manual indentation although it is worth to conduct for obtaining a wider nanostructure and faster propagating crack.

### *In situ* and complex control of cracking by indentation

**Figure 4:**
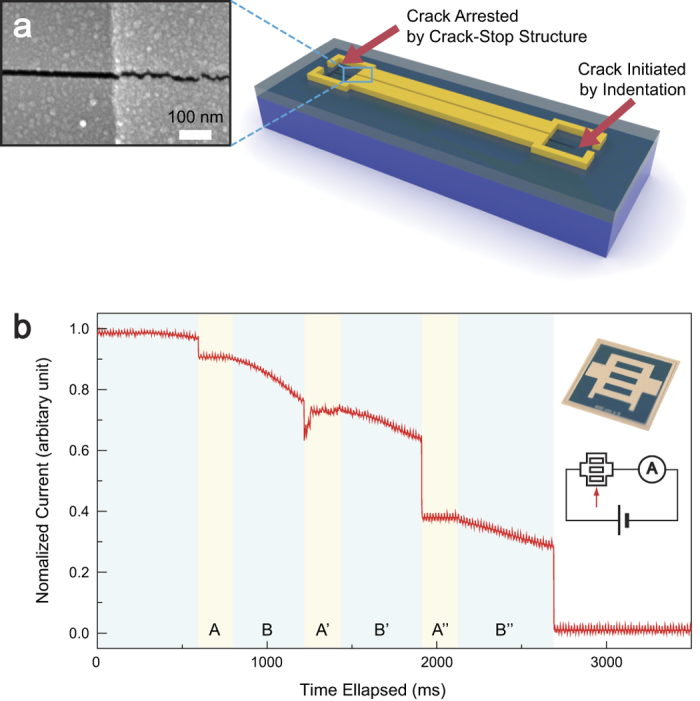
Secondary material patterning capability. (**a**) Initiated crack shearing a 15 nm thick titanium film deposited on the 800 nm thick Si_3_N_4_ cracking layer. (**b**) Crack propagation speed measurement exploiting the possibilities offered by secondary material patterning capability. The crack propagates through the lift-off patterned metal layer shown in the upper inset of (**b**) and drives changes in electrical current measured for the regions B, B′, and B″ and A, A′, and A″ which are, respectively, with and without superimposed metal layer. The lower inset is a schematic of the circuit used to measure the current change where a crack propagates to the direction indicated by the red arrow.

The nanofabrication method presented in this study differs from other nanofabrication methods, including the ones using cracking phenomena^12^,^24^, most of which employ *ex situ* fabrication. The capability of *in situ* fabrication offered by this study allows the patterning of a secondary material deposited on top of the cracking film. Figure 4a shows that following the deposition of a target material over the Si_3_N_4_ cracking medium, a crack initiated in the Si_3_N_4_/Si system propagates through the Si_3_N_4_ film and shears the superimposed target material in a pattern that matches the crack induced in the propagating medium. In effect, the crack’s propagation across the Si_3_N_4_ film serves as a template to cut the secondary material along that same crack pattern. The target layer in this particular case is a 15 nm thick titanium film deposited by e-beam evaporation, and there is no reason why other materials as a top layer in a tri-material^25^ cannot be cut as long as the energy driving the crack through the Si_3_N_4_ film is sufficient to induce the shearing of the top layer. This capacity for shearing secondary materials is likely to enable the patterning of materials which heretofore have resisted such attempts and to assist advancement in fields which especially involve biological environments^26^,^27^ and utilize nanoscale gaps^28^,^29^,^30^. With the capacity for material shearing, the availability of real-time crack creation permits the effective and precise measurement of crack propagation speed. This crack speed measurement, especially for the propagation of fast cracks, has been very difficult in the field of fracture mechanics^31^,^32^, and is an important application for the technique presented in this study. As shown in the inset to Fig. 4b, we patterned the metal layer deposited on the Si_3_N_4_ film through which a crack to be observed goes. As the cross-sectional area of the metal layer decreases, the electrical resistance presented by this layer increases throughout the crack’s propagation. To determine the true crack speed, the metal layer’s distinctive crosswalk pattern was chosen to allow the classification of the region through which the crack propagates with and without metal layers. Crack propagation is affected by material above the Si_3_N_4_ film used as a cracking template, as is clearly indicated by the values of the electrical signal shown in Fig. 4b. The particular measurements in this figure reveal that the current drops during crack propagation through a sample with a Si_3_N_4_ film thickness of 800 nm. The regions B, B′, and B″ represent the portion of the sample where the current decreases as the metal being sheared. The regions A, A′, and A″ indicate crack propagation in the absence of metal shearing; note that for these latter regions there is no current change across the bare Si_3_N_4_ film. In this particular system, the speed of crack propagation is approximately 3.1 mm/sec through the regions A, A′, A″ of 500 um in width; and it is clearly demonstrated that the speed decreases as the crack propagates beneath the metal layer in the regions B, B′, B″ (see also Supplementary Information for further discussion of the crack propagation speed measurement).

Additionally, this nanofabrication method is affected very little by the experimental environment, another attractive point for *in situ* experiments. In fact, as far as we know, this study introduces the only currently available technique that allows the fabrication processes *in situ* under an extremely broad set of circumstances, including fabrication under water (see Supplementary Information for nanochannel fabrication in aquatic environments). For the same reason, all other nanostructures presenting this study were fabricated without cleanroom facilities and conditions, and it is another unique benefit of this technique by enabling great flexibility and easiness for nanoscale experiments.

**Figure 5:**
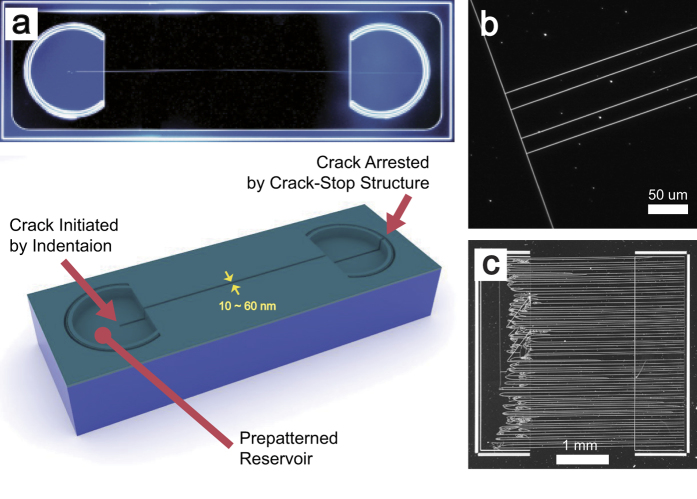
Nanochannel applications. (**a**) A complete, single-nanochannel system on a prepatterned platform containing crack-stops structure and two reservoirs fabricated using a crack initiated by indentation and arrested by a prepatterned structure. (**b**) A series of T-junctions created by a variety of nanofluidic circuits. (**c**) Rapid fabrication (60 seconds) of large number of long nanochannels without complicated equipment and skill.

Cracks generated by the indentation method introduced in this study are readily employed in the creation of 1D nanostructures. For example, this option will present significant benefits for nanofluidic research^33^,^34^, especially when long nanochannels are required for improved performance^35^. Figure 5a is an example of a 32 nm wide single nanochannel produced on prefabricated reservoirs. These reservoirs can be constructed concurrently with the creation of a crack-stop structure during the platform prefabrication etching process. Consequently, no additional processes for the functional structures are required when utilizing this simple technique. Once a prepatterned structure has been defined for a platform, various connections and shapes, as well as multiple channels, can be constructed easily whenever nanochannels are required. These platforms, designed for any number of purposes, can be mass-produced as necessary. As demonstrated by the series of T-shaped nanochannels shown in Fig. 5b, various types of nanochannel circuitries can be fabricated without difficulty. The channel junction, an essential component for nanochannel circuitry, is easily constructed through the generation of multiple crack generation perfectly perpendicular to the previously created crack, and such functional structures will be utilized directly in the nano-mixing applications^36^, especially those involving great complexity. Figure 5c shows a total of 71 32 nm-wide nanochannels connecting prepatterned reservoirs. This array of nanochannels was fabricated in an unassisted, 60-second manual manipulation on a preconstructed platform using a tungsten carbide tip. These results should indicate that the fabrication of cracks by indentation methods offers a very fast, effective, simple, inexpensive, and easy to learn approach under the conventional conditions of laboratories lacking nanofabrication facilities or even in much more rudimentary circumstances, such as those encountered during fieldwork. Moreover, the opportunity to construct multiple channels holds much promise for overcoming the limitations and difficulties^3^,^5^ that currently beset nanofabrication processes.

## Discussion

The techniques demonstrated in the present study utilize cracks to enable the fabrication of nanostructures several tens of nanometers wide with lengths limited only by the dimensions of the cracking media, while considerably reducing the problems encountered in existing nanofabrication technologies. But this method is not limited to the generation of nanochannels; through post processing and treatment, these techniques can be applied in the creation of various other 1D nanostructure constructions as well as in the material shearing and in speed sensitive *in situ* experiments. An additional strength of the new approach is the separation of the complicated preliminary platform fabrication phase from the very simple nanochannel fabrication that follows. This advantage should be apparent, for example, in the case of platforms constructed by thin film deposition to which are added, through fabrication steps including the implementation of crack-stop and other functional structures, such as reservoirs and microchannels. A user can create nanostructures with a high degree of freedom to fit the specific needs of an experimental situation without having to rely for the implementation of such structures on complex and expensive equipment.

It is important to note the poor controllability caused by unexpected factors such as crystal anisotropy and structural defects, which, however, can be managed until they are well below nuisance levels with current mature microfabrication technology during the sample preparation steps. However, careful handling may still be required for better controllability during post-processes such as the metal deposition or sample dicing processes.

## Methods

### Indentation platform fabrication

Photolithography is employed to define the sample patterns (detailed pattern example in Supplementary Information). Photoresist (AZ® 5214E, MicroChemicals) is spun on a P type (100) Si wafer initially cleaned with thin oxide removal process (10:1, H_2_O:HF) followed by Piranha cleaning for residual organic removal. Pre-defined chrome mask patterns which coincide with the sample patterns are transferred by using contact aligner (SUS MA6) and subsequent development in AZ® 300MIF. Once the photoresist pattern is defined, the Si substrate is etched by Reactive Ion Etching (Sungjin Semitech) or Deep RIE (Plasma-Therm ICP). The etch depths range from minimum of 2 *μ*m to 20 *μ*m. The Si_3_N_4_ film is deposited by LPCVD on the etched Si substrate. The deposition condition is 300 mTorr and 800 °C with source gases, DCS (dichlorosilane) and ammonia (NH_3_) of 25 and 75 sccm respectively in mass flow rate. To cut the platform in pieces, typical mechanical or laser dicing is used.

### Indentation for Crack Generation

A pencil type conical shape indenter tip coated with tungsten carbide (Korea Ace Scientific Corp.) is used for manual nanofabrication by indentation. Samples are mounted under optical microscope (SLZ series, SELOPT), and then by exerting point pressure with the indenter tip at an optimized incident angle of 30° against the platform, the nanochannel structures are characterized as the crack propagates in the Si_3_N_4_ film. The direction and the incident angle of the tip coincide with the crack propagation direction.

### Secondary Material Shearing

To test shearing capability of Si_3_N_4_ cracking, various thin film materials including Ti, Au, Pt, and Pd have been respectively deposited on the platform. Ti of 3~15 nm, Au of 10~30 nm, and Pd of 10 nm in thickness are deposited by e-beam evaporator, and Pt of 3~20 nm in thickness is deposited by sputter. All these deposited materials are patterned by two methods: lift-off and e-beam evaporator with shadow mask. The crack generation by indentation has been conducted on both Si_3_N_4_ surface and the deposited shearing material on top of the Si_3_N_4_ film.

### Real-time Nanofabrication Measurement

The metal patterns used in this study, such as inset of Fig. 4, are made by lift-off or Pt deposition using e-beam evaporator with a shadow mask. The deposited metal layer’s thickness differs with the Si_3_N_4_ thickness. Pt of 5~10 nm and 10~20 nm are deposited for Si_3_N_4_ thickness less than 800 nm and greater than 900 nm, respectably. All samples are connected in series with a reference resister, and the voltage of the reference resister is measured by an oscilloscope (DS-1530, EZ Digital). The reference resisters of resistance ranged from 0.5 to 2 kΩ have been used depending on the type of samples. As indentation placed on desired location, a crack starts to propagate. This whole process is observed under microscopic vision. Signal variations are measured by trigger function of the oscilloscope at the moment of voltage discontinuity occurrence.

## Additional Information

**How to cite this article**: Nam, K. H. *et al.* Manual, *In situ*, Real-Time Nanofabrication using Cracking through Indentation. *Sci. Rep.* **6**, 18892; doi: 10.1038/srep18892 (2016).

## Supplementary Material

Supplementary Information

Supplementary Video S1
