# Supplementary material for: Manual, In situ, Real-Time Nanofabrication using Cracking through Indentation
Source: Sci Rep. 2016 Jan 4;6:18892. doi: 10.1038/srep18892 (PMC4698748; doi:10.1038/srep18892)
Supplement: Supplementary Information [file srep18892-s1.pdf]

Supplementary Information

# Manual, *In situ*, Real-Time Nanofabrication using Cracking through Indentation

*Koo Hyun Nam*, Young Duk Suh, Junyeob Yeo and Deokha Woo*

**This Supplementary Information includes:**

6 Supplementary Discussions

6 Supplementary Figs. S1 to S6 with captions.

1 Supplementary Video S1

## I. Crack stop structure

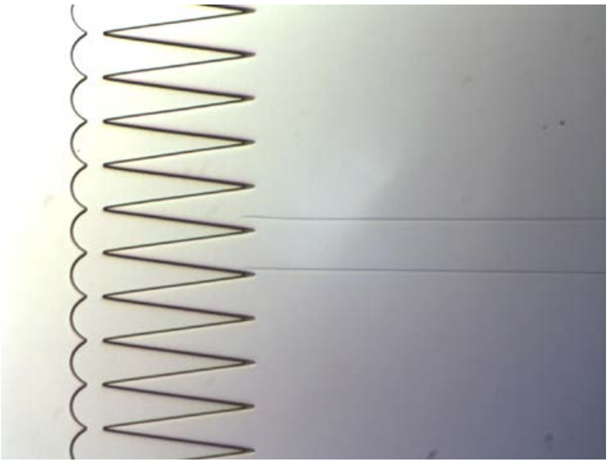

**Figure S1.** Image of microfabricated notches in the Si substrate captured by an optical microscope. These structures are used to arrest cracks approaching to the area protected by the notches.

Cracks in brittle $Si_3N_4$ film propagate energetically, and complete stops are very difficult to achieve in most circumstances. Thus, in addition to using crack-stop structure to tailor nanochannels specific applications, our approach can also protect platforms against external crack invasion likely to occur during dicing process. A few techniques which have been devised to arrest cracking[1], but none of them approach the techniques demonstrated in this study in terms of either reliability or applicability.

As shown in Fig. S1, for this work we positioned notch structures, originally employed for crack initiation, to arrest propagating cracks. The geometry effects in the vicinity of the notch structures generate a distortion of the residual stress of the $Si_3N_4$ medium, and the crack stops as it enters the region of the altered residual stress below the amount required to continue a crack to propagate. Additionally, careful design and controlled fabrication conditions make the initiation of a crack from the notch structure very unlikely when the thickness of the $Si_3N_4$ falls below a certain level. Along with the cracking stability discussed in the main text, this factor determines the thickness of $Si_3N_4$ deposition for platform fabrication.

## II. Easiness of nanoscale pattern fabrication

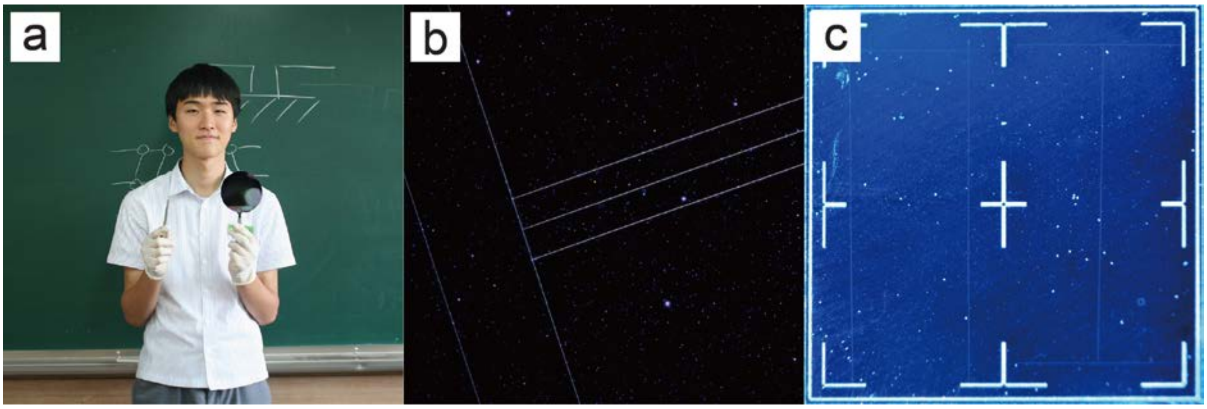


**Figure S2.** A high school student with no prior experience has received simple instructions for the inscription through manual indentation of a sample with complex nanostructures. a) The student, Sangyoon Yoon, fabricated the nanostructures with the tungsten carbide pencil and the $Si_3N_4$/Si platform at his school. b) T-junction nanochannel easily fabricated after a little practice by Mr. Yoon. c) A more complicated sample with the letters, “NI,” (representing the initials of his school's name, “Neung-In”) fabricated by manual indentation. These letters, several millimeters long, along with the sample shown in Fig. 1d which uses the same sample platform, constitute one of the world's longest nanometer-wide letter inscriptions.

## III. Optical observation of nanochannel and its practical usages

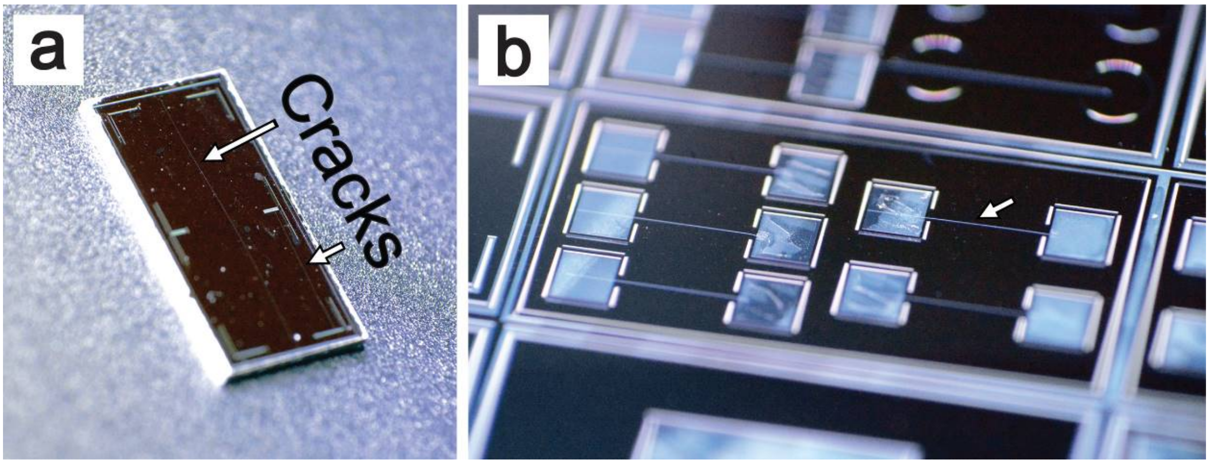


**Figure S3.** Nanoscale crack imaged by a general-use digital camera (NIKON D5200) under strong illumination. a) Two straight cracks are generated on a diced platform. b) A number of nanochannels are generated to connect pairs of square reservoirs for nanofluidic experiments. These reservoirs are functional structures prefabricated on the silicon wafer, and the nanostructures can be fabricated on demand any time later. These nanoscale patterns are even clearer when observed by the naked eye. The widths of these channel-like nanostructures are much smaller than the smallest optically visible objects as determined by the diffraction-limit of imaging system. The visibility of the nanostructures is thought to result from the light scattering at the sharp corners of the crack cleavage. The unaided-eye visibility of this nanofabrication process is a key factor making this technique a unique manual nanoscale pattern generation methodology.

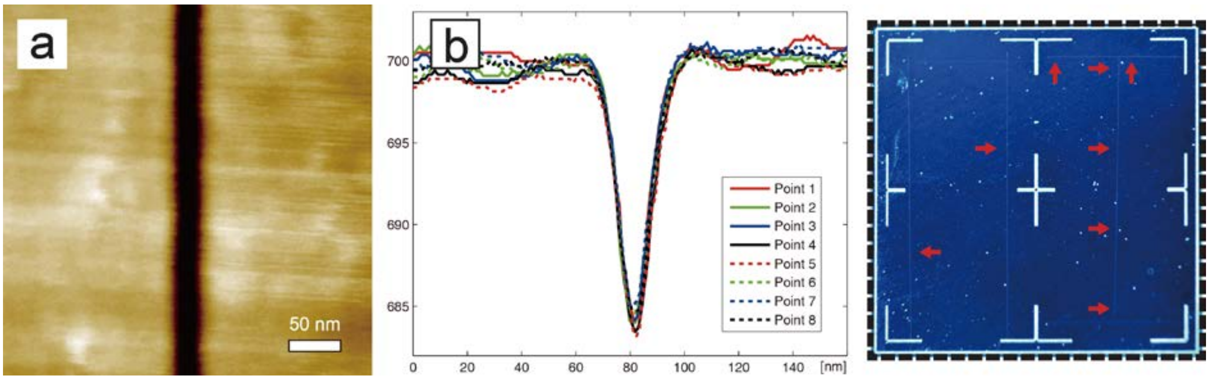


**Figure S4.** Atomic force microscopy (AFM) observations which verify the uniformity of the width of the crack. a) The surface profile shows a uniform, straight crack generated vertically in the middle of the figure. b) AFM surface profiles (non-contact mode) of the cracks at eight different locations as indicated with arrows in the figure inset (right).

# IV. Substrate penetration of channeling cracking

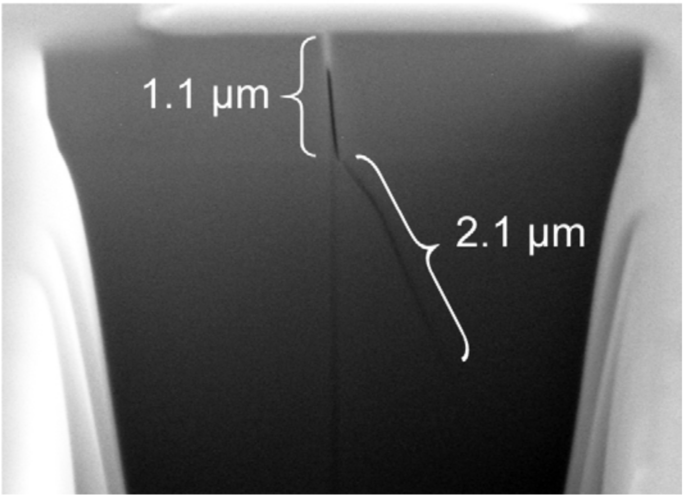


**Figure S5.** SEM image of FIB-milled sample showing crack penetration into the substrate extended from the crack in the $Si_3N_4$ film.

The theoretically derived crack depth in the substrate is given by $d_s = (\Omega_c \Sigma - 1)(\Gamma_f / \Gamma_s) h_f$, where $\Sigma$ and $\Gamma_s$ are the dimensionless elastic mismatch number and the fracture energy of the substrate, respectively[2, 3]. Numerical calculation for our $Si_3N_4$ film/Si substrate system shows substrate penetration of 2.01 µm, and this is in good agreement with the experiment results, and one of which is shown in Fig. S5.

Compared to the cracks that resulted in the substrate penetration in Fig. S5, the crack in Fig. 2b has its tip stopped at the interface between the film and substrate. In the case of fully cracked film without substrate penetration, the stress intensity factor is defined by $K_I = \sigma(\pi h)^s f$, where $s$ is a stress singularity exponent, and $f$ is a nondimensionalized stress intensity factor[2]. Both variables are defined by material properties. As our experiment results show, the substrate penetration is not observed in the samples of thin $Si_3N_4$ film. The increase of the thickness ($h$) of the $Si_3N_4$ film raises the stress intensity factor, and the substrate penetration into the Si wafer occurs when the stress intensity factor exceeds the fracture toughness of the Si. The transition thickness of $Si_3N_4$ film is found to be 900 nm, and we started to observe the substrate penetration among the samples for $Si_3N_4$ film with thickness ranging between 600 to 1100 nm. Note that the propagation speed of cracks dramatically increases in the similar region of the thickness range. We anticipate that the substrate penetration relaxes the constraint of the cracking material, which, in turn, lowers energy to continue cracking[3]; thus, the surplus energy contributes more to its dynamics.

# V. The electrical measurement methodology of crack propagation speed

Propagation of cracking in the metal deposited region as shown in the inset of Fig. 4b is affected by the metal

layer, and a similar effect could be found in the area closed to the interface between the regions with and without metal deposition. To minimize this effect, the thickness of the metal layer should be small, but it should be noted that the resistance of the metal layer increases accordingly. This crack speed measurement method was easily conducted without fastidious sensitivity to the type and the thickness of metal deposited. Due to different scales of propagation speed, each sample with different thicknesses of $Si_3N_4$ was conducted with several different configurations of metal layers and corresponding measurement systems. The metal patterns used for this experiment, such as the inset in Fig. 4b, are made by lift-off or deposition with a shadow mask. The deposited metal layer's thickness differs with the $Si_3N_4$ thickness. Pt layers of 5 ~ 10 nm and 10 ~ 20 nm are deposited for $Si_3N_4$ thickness values less than 800 nm and greater than 900 nm, respectively. All samples are connected in series with a reference resister, and the voltage of the reference resister is measured with an oscilloscope (DS-1530, EZ Digital). Reference resisters of resistance ranging from 0.5 to 2 kΩ have been used depending on the sample type. As indentation placed on desired location, a crack starts to propagate. This whole process is observed under microscopic vision. Signal variations are measured by trigger function of the oscilloscope at the moment of voltage discontinuity occurrence.

# VI. Nanochannel fabrication in aquatic environment

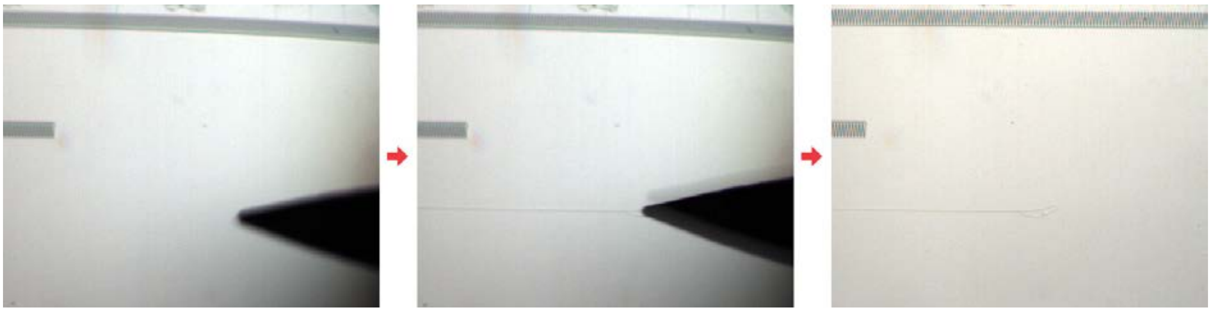

**Figure S6.** Optical microscopic images of sample being indented showing crack initiation and propagation under tap water.

The majority biology and life science experiments are conducted in aquatic environments including the circumstances under liquid solutions. Thus, the complications presented by these experimental conditions must be overcome for *in situ* fabrication to be effective. There are few nanofabrication methodologies that can be undertaken in such environments, even when system dimensions are expanded to the microscale. Figure S6 shows that cracks initiated by indentation in tap water propagate as effectively as those that are initiated in open air.

As shown in the left image of Fig. S6, the entire sample can be immersed into tap water when indentation is applied to initiate a crack. The middle image in Fig. S6 confirms that there is no effect of the surrounding water environment on the propagation of a crack. The distortion of the images shown in the left and middle image in

Fig. S6 is recovered when the indenter tip is removed from the water, as shown in the right image in the same figure.

Therefore, crack-induced nanofabrication is affected only negligibly by the surrounding environment, and should be useful in a number of applications that require experimental environments which do not allow clean rooms or carefully regulated and maintained conditions of temperature and pressure.

## Supplementary Video

**Supplementary Video S1.** Complete real-time viewing of crack initiation and propagation through a $Si_3N_4$ film of 700 nm in thickness on a Si substrate. The speed of crack propagation is approximately 600 μm/sec.

## Supplementary References


S1. D. Green, R. Tandon, V. Sglavo, Science 1999, 283, 1295; M. P. Rao, A. Sanchez-Herencia, G. Beltz, R. McMeeking, F. Lange, *Science* **286**, 102 (1999).

S2. J. Beuth Jr, *Int. J. Solids Struct.* **29**, 1657 (1992).

S3. T. Ye, Z. Suo, A. Evans, *Int. J. Solids Struct.* **29**, 2639 (1992).
